# Opportunities and Challenges of Backbone, Sidechain, and RDC Experiments to Study Membrane Protein Dynamics in a Detergent-Free Lipid Environment Using Solution State NMR

**DOI:** 10.3389/fmolb.2019.00103

**Published:** 2019-10-25

**Authors:** Stefan Bibow

**Affiliations:** Biozentrum, University of Basel, Basel, Switzerland

**Keywords:** RDC, NMR, Carr-Purcell-Meiboom-Gill (CPMG), CEST, membrane protein dynamics, backbone, side chain

## Abstract

Whereas solution state NMR provided a wealth of information on the dynamics landscape of soluble proteins, only few studies have investigated membrane protein dynamics in a detergent-free lipid environment. Recent developments of smaller nanodiscs and other lipid-scaffolding polymers, such as styrene maleic acid (SMA), however, open new and promising avenues to explore the function-dynamics relationship of membrane proteins as well as between membrane proteins and their surrounding lipid environment. Favorably sized lipid-bilayer nanodiscs, established membrane protein reconstitution protocols and sophisticated solution NMR relaxation methods probing dynamics over a wide range of timescales will eventually reveal unprecedented lipid-membrane protein interdependencies that allow us to explain things we have not been able to explain so far. In particular, methyl group dynamics resulting from CEST, CPMG, ZZ exchange, and RDC experiments are expected to provide new and surprising insights due to their proximity to lipids, their applicability in large 100+ kDa assemblies and their simple labeling due to the availability of commercial precursors. This review summarizes the recent developments of membrane protein dynamics with a special focus on membrane protein dynamics in lipid-bilayer nanodiscs. Opportunities and challenges of backbone, side chain and RDC dynamics applied to membrane proteins are discussed. Solution-state NMR and lipid nanodiscs bear great potential to change our molecular understanding of lipid-membrane protein interactions.

## Introduction

In 1985, the first membrane protein (MP) structure was determined (Deisenhofer et al., 1985). Since then, our knowledge of protein structure and function increased dramatically and it is now clear that a mechanistic understanding of protein function requires a comprehensive understanding of both protein structure and dynamics. For membrane proteins the function-dynamics relationship is important to explain gating, transport (allosteric), signal transduction, or biased signaling. Despite tremendous efforts and progress, detailed insights into the function-dynamics relationship of MP dynamics remains a challenging and tedious endeavor. A major bottleneck is still the difficulty to obtain atomic-resolution data of membrane proteins in lipids. X-ray crystallography has provided by far the most membrane protein structures (Bill et al., 2011) and there has been considerable progress by cryo-electron microscopy (cryo-EM) to provide high-resolution structural data. Both techniques, however, require cryogenic temperatures that provide static “snapshots” of dynamic processes. As an alternative, solution state NMR has established itself as a method to provide structural and dynamics data for the structural biology of membrane protein. Moreover, the recent advancements in higher-field NMR spectrometers, specific isotope labeling (Tugarinov et al., 2006; Gans et al., 2010; Bellstedt et al., 2013), new TROSY-type experiments (Lakomek et al., 2012, 2013) and new lipid-bilayer nanodisc assemblies (Bayburt et al., 1998; Gluck et al., 2009; Knowles et al., 2009; Dorr et al., 2014) opens exciting new possibilities to study the function-dynamics relationship of MPs in lipids without detergents and the need of crystallization, freezing or sedimentation. Before the dawn of nanodiscs lipid-detergent bicelles provided a viable alternative to detergent micelles in order to study membrane proteins in a lipid environment. However, recent results suggest that bicelle preparations with q-values below 1 likely represent an environment where lipids and detergent molecules mix (Caldwell et al., 2018). Those low-q bicelle preparations are necessary for solution NMR to obtain sharp and interpretable NMR spectra since they retain a fast tumbling of the membrane protein. In contrast, MPs in detergent-free nanodiscs are fully embedded in a lipid environment, reasonably-sized for solution NMR, monodisperse, highly reproducible and suitable for a wide range of biophysical methods and assays.

The original nanodisc is composed of a so-called membrane scaffold protein (MSP). MSP possesses amphipathic helices and two copies of MSP wrapped around a lipid bilayer patch to form a barrier between the hydrophobic interior (lipids) and hydrophilic exterior (solvent). MSP is a shortened or elongated version of the human ApoA-I protein that is the major scaffolding protein of discoidal and spherical HDL particles (Jonas, 1986; Bayburt et al., 1998; Bibow et al., 2017). The size of MSP-nanodiscs is independent of the used lipids or incorporated MP and can be conveniently controlled via the length of MSP (Denisov et al., 2004). That means that properly assembled MP-containing MSP-nanodiscs will usually always come at a similar position on the size-exclusion chromatogram (SEC), ideally exhibiting a symmetric peak, which can serve as a first quality check for the assembly. For example, tailing of the peak indicates that either too many or too few lipids were used during the assembly process when the tailing happens at the beginning or at the end of the peak, respectively. Visualization of the SEC peak fractions on a SDS gel can then be used to evaluate and optimize MP incorporation efficiency using different lipids, different ratios or different (usually lower) concentrations of the molecules. The peak fractions can be pooled and either measured directly or subjected to an Nickel-Immobilized Metal Affinity Chromatography (Ni-IMAC) to separate empty MSP-nanodiscs from nanodiscs containing a purification-tagged MP. Importantly, the solution is devoid of MP-detergent complexes due to the use BioBeads (Biorad) that adsorbs any detergent from the solution. Hence, MPs not incorporated in lipid-bilayer nanodiscs will precipitate out of the solution.

Ten years ago, an additional detergent-free discoidal nanodisc was developed using styrene maleic acid (SMA) (Knowles et al., 2009). SMA nanodiscs are also known as SMALPs and the polymers are commercially available under name Lipodisq (e.g., Sigma). SMA has more efficient solubilization properties than ApoA-I. That allows to solubilize biological membranes to extract membrane proteins without the use of detergents. Conveniently for solution NMR, SMA forms monodisperse discoidal particles with a diameter of around 9–12 nm (Orwick et al., 2012; Scheidelaar et al., 2015). Inconveniently, however, the size of the SMA nanodiscs cannot be controlled as it depends on the incorporated membrane protein and can be as large as 24 nm in diameter (Scheidelaar et al., 2015). Recently, Ravula et al. synthesized an amine modified SMA derivative called SMA-EA (Ravula et al., 2017b). The size of those nanodiscs can be tuned between 10 and 50 nm, depending on the lipid-to-polymer ratio. Subsequently, the same group synthesized another derivative, called SMA-QA (styrene maleimide quaternary ammonium) that are extremely stable over a wide range of divalent ion concentrations and between a pH of 2.5 to 10 (Ravula et al., 2017a, 2018). Notably, the larger nanodiscs, called macro-nanodiscs by the authors, align in the external magnetic field (Ravula and Ramamoorthy, 2019), similar to the alignment of previously prepared 30 nm macrodiscs (Park et al., 2011). Both macrodisc preparations can be used to align membrane or soluble proteins to extract residual dipolar couplings (see below) for NMR studies. Apart from NMR, also other biophysical methods, such as microscale thermophoresis, Bio-layer Interferometry or surface plasmon resonance spectroscopy (SPR) benefit from a detergent-free MP reconstitution. In SPR for example, a detergent-containing flow buffer must be used to replenish detergent molecules from the micelle surrounding the MP bound to the sensor chip (Kaur et al., 2019).

**Figure 1:**
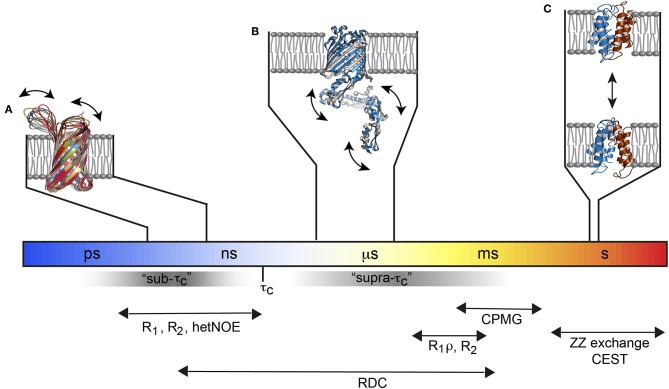
Functional membrane protein dynamics and selected NMR experiments. **(A)** The loops of the Outer membrane protein X (OmpX) are flexible and move on the ps–ns timescale (PDB: 2MNH). **(B)** The μs timescale dynamics of the POTRA domains from BamA (PDB: 4k3b) are invisible for most of the standard NMR relaxation experiments since they fall in the “hidden” supra-τ_c_ window. **(C)** The multidrug transporter EmrE alternates between the outward- and inward-open state about 5 times per second (PDB: 3B5D). Figure modified with permission from Bibow and Hiller (2018).

Solution NMR methods that extract dynamics parameters are very well-developed in the sense that they can provide dynamical insights into basically every atom of an amino acid on a broad range of timescales from picoseconds to seconds with high precision (Fischer et al., 1998; Jarymowycz and Stone, 2006; Kleckner and Foster, 2011; Morin, 2011; Palmer, 2014; Liang and Tamm, 2016; Ishima and Bagby, 2018; Stetz et al., 2019). Picoseconds to nanosecond motions, i.e., motions that are faster than the molecular tumbling time τ_c_, can be studied by nuclear spin relaxation experiments. This is sometimes called the sub-τ_c_ range. Slow timescale motions are probed by relaxation dispersion experiments. There, stochastic fluctuations (that are independent of rotational tumbling) between different chemical environments increases the line width of affected resonances in the NMR spectrum and reduces the signal intensity which is exploited by the above-mentioned dispersion experiments. However, the stochastic fluctuations must be slower than ca. 50 μs to be measurable. That creates an invisible time window between τ_c_ and ca. 50 μs, sometimes referred to as the “hidden” supra-τ_c_ window (Figure 1). Residual dipolar couplings (RDCs) are independent of the τ_c_ of the molecule, which is why they can detect motions faster and slower than τ_c_ up to the same limit that is used for NMR relaxation dispersion measurements. Thus, RDCs are sensitive to the “hidden” supra-τ_c_ window. The measurement of RDC dynamics therefore provides direct access to protein motions on timescales that complements the dynamic picture derived from nuclear spin relaxation and relaxation dispersion measurements (Figure 1).

Nanodiscs and solution NMR are a very powerful combination in providing structural as well as dynamics information with atomic-resolution for the membrane protein itself and also for the surrounding lipids (Brainard et al., 1984; Shaw et al., 2004; Mors et al., 2012; Frey et al., 2018). These new opportunities finally enable to inter-correlate the structural and dynamics response of MPs and lipids upon variation of temperature, lipids and other environmental factors at atomic resolution within the same sample (Frey et al., 2017, 2018). It is therefore surprising that there are still only a few solution state NMR publications that investigate MP backbone dynamics in a lipid environment. This is certainly a result of at least three aspects: (i) the difficulty to work with membrane proteins; (ii) the difficulties arising from the usage of bicelles; (iii) the recency of smaller nanodiscs that can provide high-quality spectra in a very reproducible manner.

This review is therefore a synopsis of recent developments in exploring the dynamics landscape of MP in lipids using solution NMR. Due to the fact that no RDC or sidechain CPMG dynamics studies of MP in lipids were available, studies using soluble proteins or membrane proteins in detergents will be reviewed that can provide starting points to guide future studies. I also provide preliminary data of ^1^H methyl-CPMG relaxation dispersion experiments from OmpX in lipid nanodiscs that show a complex temperature and membrane position dependence. I subsequently discuss the opportunities and problems of using side chain and RDC dynamics.

## Backbone Resonances as Probes for Membrane Protein Dynamics

By far the most frequently studied protein dynamics are those from the protein backbone, more specifically the ^15^N dynamics of the ^1^H-^15^N bond (Kay et al., 1989; Wagner, 1995). Fast timescale dynamics are traditionally investigated by nuclear spin relaxation experiments. Local magnetic fields are generated by the amide ^1^H-^15^N dipolar interactions and the ^15^N chemical shift anisotropy (CSA) of the ^1^H-^15^N bond. Nuclear spin relaxation emanates from the temporal fluctuations of the local field in amplitude and direction due to molecular tumbling and internal bond dynamics. Nuclear spin relaxation therefore provides direct insights into the reorientational motion of the ^1^H-^15^N bond vector. In the most commonly applied approach, the so-called model-free approach (Lipari and Szabo, 1982a,b), bond vector motions are separated into internal dynamics that occur on the picosecond timescale and the overall molecular tumbling τ_c_ of the macromolecule, which is typically in the range of nanoseconds. The model-free approach results in the extraction of three parameters: the overall tumbling time τ_c_; the spatial restriction of a given internuclear bond-vector (denoted as the Lipari-Szabo order parameter $S_{LS}^{2}$); and the local internal correlation time τ_e_ (typically in the 10's−100's of ps) of that residue. In the extended model free formalism (Clore et al., 1990) it is assumed that internal motions occur on two separate timescales with τ_f_ < 20 ps and τ_s_ > 500 ps (Jarymowycz and Stone, 2006). The Lipari-Szabo order parameter $S_{LS}^{2}$ ranges from 0 to 1. In the most frequently used “diffusion-in-a-cone” model, the bond vector motion is interpreted to diffuse freely within a cone. The cone angle is related to $S_{LS}^{2}$ in the sense that when the cone angle of the bond vector decreases from 90° to 0° (tantamount to an increasing spatial restriction of internal bond-vector motions), $S_{LS}^{2}$ increase from 0 to 1.0. For the model-free approach, typically longitudinal (*R*_1_) and transverse (*R*_2_) relaxation rate constants as well as the heteronuclear steady-state nuclear Overhauser effect (hetNOE) are measured from a series of 2D ^1^H-^15^N correlation spectra at one or more magnetic field strengths (Kay et al., 1989; Jarymowycz and Stone, 2006). In one study the backbone dynamics of the β-barrel membrane protein OmpA reconstituted in DPC micelles were measured at three magnetic fields and analyzed using the extended model-free formalism (Liang et al., 2010). It was found that the internal motions of residues near the center of the β-barrel are highly restricted (high $S_{LS}^{2}$), but increase toward the barrel ends. Loops are flexible and move with progressively larger amplitudes once they emerged out of the micelle (Liang et al., 2010). Liang et al. noted that no clear trend could be identified whether one or two time scales of internal motions should be used to describe the relaxation data. It might be, that the model free formalism runs into its limitations because of the complex detergent dynamics. For example, in the case of OmpA, the central β-barrel residues would be in contact with the very flexible acyl chain ends of the detergent, whereas the aromatic-rich ends of the β-barrel are in contact with the less-flexible polar headgroups of the detergent. Furthermore, loop residues are exposed to the solvent, which exhibits yet another dynamic environment. Not only is the chemical environment different in these different regions of the protein, but the detergent itself possesses and experience a dynamic gradient from the lipid–water interface into the hydrophobic core (Brown et al., 1983; Brainard et al., 1984). Hence, MPs, such as OmpA experiences motions that depends on the position of the residue within the detergent environment and that differ significantly from those of globular proteins, for which the formalism was originally developed. Notably, the model-free formalism has not yet been applied to extract internal dynamics from a membrane protein surrounded by lipids. Although a similar dynamic gradient is likely, it will be interesting to see what impact a stronger hydrophobicity has on the internal dynamics of β-barrel and α-helical membrane proteins.

The exchange of amino acids of a protein between different chemical environments give rise to an additional relaxation contribution called chemical or conformational exchange. Since exchange phenomena take place on a timescale slower than the overall tumbling time τ_c_, they don't affect R_1_, but R_2_. Exchange phenomena probably provide the most informative and interesting data since catalytic events, gating and other MP signal transductions take place in the μs to ms timescale. Residues that experience chemical exchange with exchange rates between 50,000 and 100 s^−1^, and conformation lifetimes between around 50 μs and 10 ms have reduced NMR signal intensities and broadened linewidths. The observed R_2_ (R_2,eff_) is then a sum of the exchange-free transverse relaxation rate (often denoted $R_{2}^{0}$, dominated by dipole-dipole and CSA relaxation as mentioned above) and the exchange contribution R_ex_. Because R_ex_ (and hence R_2_) depends on radiofrequency (rf) pulses applied during the NMR pulse sequence, experimental methods aimed at quantifying conformational exchange apply variable rf fields (rf pulses) during the NMR experiment that enable detailed characterization of structures, kinetics, and equilibria of interconverting species, even for conformations that are populated only a few percent (Palmer, 2014). In the case of conformational exchange with exchange rates between 1 and 100 s^−1^, and conformation lifetimes between 1,000 and 10 ms, peaks for each conformation can become visible in an NMR spectrum. So called “peak doubling” can be an indicator for slow conformational exchange (but also for impurities). If resonances are visible for each conformation, the ZZ exchange experiment can be measured with different mixing times (Farrow et al., 1994; Li and Palmer, 2009). Several ZZ exchange experiments with different exchange times provide built-up curves for cross-peaks that connect the same residue in different chemical environments, i.e., different conformations (Palmer, 2014). Analyzing cross- and diagonal-peak intensities allows to extract exchange rates and the associated populations, for example between the outward- and inward-open states of the small multidrug transporter EmrE (see below) (Morrison et al., 2011). The ZZ exchange experiment measured with one mixing time can already answer the question which peaks belong to the same amino acid due to the presence of the connecting cross-peak. If one conformation is very lowly populated, only the peakset for the higher populated state might be visible, despite being within the slow-exchange regime. In such a specific case, the recently developed Chemical Exchange Saturation Transfer (CEST) experiment can directly reveal exchange rates, the chemical shifts of the sparsely populated conformation and, in ideal cases, their R_2_ values (Vallurupalli et al., 2012, 2017). To the knowledge of the author, there is currently no CEST study on a membrane protein.

In the case were one single and broadened resonance peak for a given residue is observed, two approaches exist that measure the dispersion of transverse magnetization. In one approach, conformation lifetimes as short as ca. 50 μs can be routinely assessed using the R_1ρ_ experiment, were the relaxation dispersion is measured as a function of the effective field in the rotating frame by varying the rf amplitude or frequency. In the CPMG (Carr-Purcell-Meiboom-Gill) experiment, a train of 180° pulses is applied during the relaxation delay with variable inter-pulse delays. Motions with lifetimes of 300 μs to 10 ms (corresponding to conformational exchange rates *k*_ex_ = 100–3,000 s^−1^) can be quantified with CPMG (Kleckner and Foster, 2011; Bibow and Hiller, 2018). CPMG experiments were conducted to investigate dynamics of the β-barrel protein PagP that transfers the *sn*-1 palmitate chain from phospholipid to lipopolysaccharide in Gram-negative bacteria (Hwang et al., 2004). The authors refolded PagP in the detergent CYFOS-7 which is similar to DPC except that it possesses a bulky cyclohexyl ring at the terminus of its alkyl chain. The decision for this rather unusual detergent was based on the X-ray structure which showed the presence of a single inhibitory LDAO molecule in the active site, and it was rationalized that the bulky acyl chain may not enter the active side. Indeed, PagP was found to retain catalytic activity in CYFOS-7, unlike all the other detergents that had been used in previous studies (Hwang and Kay, 2005). The CPMG experiments revealed exchange rates of *k*_ex_ = 331 ± 40 s^−1^ between the visible R (relaxed) state and the “invisible” T (tense) state that was quantified to be populated around 10% at 45°C (Hwang et al., 2004). Upon lowering the temperature to 25°C, the exchange was slowed down significantly. This and the fact that the “invisible” state was higher populated at lower temperature lead to additional peaks in the spectrum from the previously invisible state. Using ZZ exchange experiments, the authors could determine the exchange kinetics with average values of the R → *T*(*k*_R _→_ T_) and T → R (*k*_T_→_ R_) conversion rates of 2.8 ± 0.5 s^−1^ and 6.5 ± 0.9 s^−1^, respectively, and a fractional population of the T state to be around 30% at 25°C.

I mention the two MP-detergent studies because they are prime examples that exploit the diversity and potential of relaxation experiments and because there are only very few examples that investigate MP dynamics in nanodiscs and other lipid scaffolding assemblies. Nanodiscs are around since the 1980's (Brainard et al., 1984; Bayburt et al., 1998; Lyukmanova et al., 2008; Gluck et al., 2009). Their full potential, however, could only recently be harvested for membrane protein structure determination (Hagn et al., 2013; Bibow et al., 2014) and dynamics studies (Brewer et al., 2011; Mineev et al., 2015; Frey et al., 2017, 2018; Lakomek et al., 2017; Ueda et al., 2019). They are able to provide information with atomic-resolution not only for the membrane protein itself but also for the surrounding lipids. The complexity and impact of the environment on MP dynamics was recently investigated using the β-barrel membrane protein OmpX reconstituted in n-dodecyl-phosphoscholine (DPC) micelles, dihexanoyl-phosphatidylcholine (DHPC):di-myristoyl-phosphatidylcholine (DMPC) bicelles and in DMPC nanodiscs (Frey et al., 2017). OmpX retains is structure in micelles formed from DHPC (Fernandez et al., 2001) and DPC (Hagn et al., 2013), DHPC/DMPC bicelles (Lee et al., 2008) and DMPC:DMPG (ratio of 3:1, DMPG, dimyristolyphosphatidylglycerol) lipid nanodiscs (Hagn et al., 2013; Bibow et al., 2014), making it the ideal model system to assess the impact of the environment. Chemical shift differences between the DPC detergent and DMPC and DMPC/DMPG lipid environment revealed that the detergent and lipid exhibit a different chemical environment. In contrast, OmpX chemical shifts between DMPC bicelles and DMPC/DMPG nanodiscs overlapped, demonstrating that the bicelle formation was successful and that OmpX is surrounded largely by DMPC in bicelles and nanodiscs (Frey et al., 2017).

**Figure 2:**
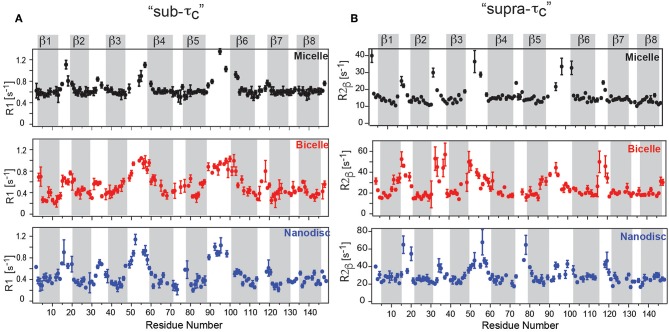
OmpX membrane protein dynamics in different membrane mimetics. **(A)** “sub-τ_c_” dynamics measured using R_1_ relaxation revealed a flat profile for OmpX in micelles but dynamic “ramps” in nanodiscs and bicelles between β-strands 3, 4, 5, and 6 and the flexible extracellular loops. **(B)** “supra-τ_c_” dynamics, measured using the R_2β_ experiment shows a gradual transition between rigid β-strand and flexible loop dynamics in bicelles and nanodiscs due to conformational or chemical exchange in the μs-ms timescale. Notably, the gradual transition is largely absent in micelles. Figure reproduced with permission from Frey et al. (2017).

The measurement of molecular tumbling using TRACT (Lee et al., 2006) revealed a τ_c_-value of ca. 22 ns for the DPC micelle, ca. 40 ns for OmpX in nanodiscs and ca. 35 ns for OmpX in the bicelle. Fast motions on the ps–ns timescale were obtained using *R*_1_ and hetNOE experiments, while slow timescale motions in the μs–ms range were obtained from *R*_1_ρ (υ_SL_ = 2,000 Hz) and *R*_2_ experiments (Frey et al., 2017). Fast timescale motions revealed high hetNOE values of around 0.8 in all three assemblies, in agreement with a previous study (Hagn et al., 2013). *R*_1_ rates for the rigidly formed β-strands were around ca 0.6 s^−1^ for the micelle and 0.3–0.4 s^−1^ for the bicelle and nanodisc, reflecting the faster tumbling for the micelle (Figure 2A). The rigidly formed β-strands were interrupted by flexible loops exhibiting higher *R*_1_ rates and low hetNOE values of around 0.4. The 8 β-strands of OmpX could therefore be identified clearly in all three assemblies (Frey et al., 2017). Surprisingly, the dynamic variability differed strongly between the lipid and detergent environment. Whereas, dynamic “ramps” are observed in nanodiscs and bicelles between β-strands 3, 4, 5, and 6 and the flexible extracellular loops, OmpX in micelles exhibits a flat profile for its β-strands, sharply interrupted by flexible loops (Figures 2A,B). The gradual transition with increasing R_2β_ values between β-strands and loops in nanodiscs and bicelles is found to be a result of exchange in the μs-ms timescale, that is not present in the detergent micelle (Frey et al., 2017) (Figure 2B). Taken together, the study revealed a pronounced dynamic variability on the pico- to nanosecond and micro- to millisecond timescale for the β-strands in the lipid environment, which appears to be suppressed in DPC micelles. This behavior suggests that the frequent loss of membrane protein activity in detergents might be related to reduced/altered dynamics and that lipid flexibility appears to play an important role in MP activity. DPC has been the subject of some controversy recently (Chipot et al., 2018). Some reports have indicated that DPC can alter the structure and dynamics of membrane proteins and their interaction interface with ligands (Zoonens et al., 2013; Dehez et al., 2017). The above-mentioned study by Frey et al. therefore supports the view that dynamics studies in DPC must be evaluated with particular care.

**Figure 3:**
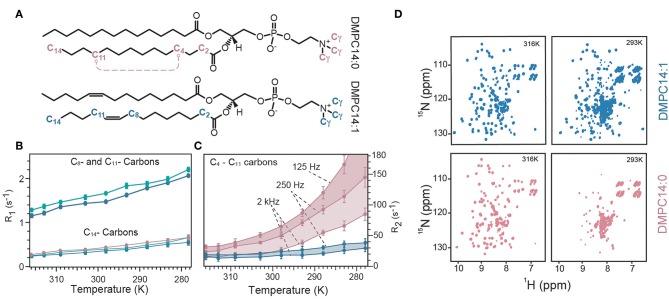
Temperature-dependent lipid and OmpX dynamics. **(A)** Representation of the used lipids either with its saturated acyl chain (termed DMPC14:0) or 9-cis unsaturated acyl chain (termed DMPC14:1). **(B)** R1 relaxation rates of C14, C8, and C11 carbon atoms of the lipid acyl chain are plotted against the temperature for DMPC14:0 (red), DMPC14:1 (blue), and DMCP14:1 with cholesterol (green). Addition of cholesterol to DMPC14:1 increases R1 rates of C8 and C11. **(C)** R2 relaxation rates for the C4–C11 carbons of the acyl chain measured with CPMG-frequencies of 125, 250, and 2,000 Hz. A CPMG-frequency of 125 Hz probe ms motions which become significant below the T_m_. The R2 rate for DMPC14:0 at 278 K was 242 ± 20 s^−1^, with a CPMG-frequency of 125 Hz but was excluded for visibility reasons [same color-code as in **(B)**]. **(D)** The spectral quality of OmpX were used as a qualitative measure to assess ms dynamics. At 316 K spectra of similar quality can be acquired for DMPC14:0 and DMPC14:1, whereas at 293 K the spectrum of DMPC14:0 is significantly worse than for DMPC14:1. Figure reproduced with permission from Frey et al. (2018).

The observed strong and membrane mimetic-dependent dynamic behavior revealed an unexpectedly powerful influence of the lipids on MP dynamics. In a follow-up study, the authors examined the influence of lipid dynamics in more detail. They reconstituted OmpX in three different lipid environments composed of either a saturated lipid (1,2-dimyristoyl-sn-glycero-3-phosphocholine, termed “DMPC14:0”), unsaturated lipid (cis-9-tetradecanoyl-sn-3-glycero-3-phosphocholine, termed “DMPC14:1,” Figure 3A). Furthermore, OmpX was also reconstituted in cholesterol-containing unsaturated lipid bilayers to assess the influence of different lipid phases, because the addition of cholesterol to the unsaturated DMPC14:1 bilayer converts the liquid-disordered lipid phase to a liquid-ordered phase (Frey et al., 2018). The influence of membrane fluidity (or lipid order) on membrane protein dynamics was first investigated by temperature-dependent NMR measurements on lipids since the degree of fluidity can be manipulated by temperature. By using the ^13^C inversion recovery experiments, R_1_ relaxation rates of lipid carbons at different temperatures were determined (Figure 3B). Increasing relaxation rates upon addition of cholesterol and temperature reduction reveals that ^13^C R_1_ relaxation rates of acyl chains probe motions in the in the 100's of picoseconds. That corroborated the idea that lipid R_1_ rates report on trans–gauche isomerization motions that are on the time scale of hundreds of picoseconds and are influenced by cholesterol (Frey et al., 2018). ^15^N R_1_ relaxation measurements on OmpX revealed a dynamic coupling in the ps–ns time scale between the lipid environment and the immersed membrane protein OmpX. The authors noted that the results indicate that motions faster than 2.65 ns are imposed on the rigid β-strand residues since R_1_ changes upon temperature variations are specific to the lipid phases which exhibit different lipid trans–gauche isomerization rates (Frey et al., 2018). Subsequently, ^13^C CPMG experiments with CPMG-frequencies of ν_cpmg_ = 250, 2,000, and 125 Hz were recorded to extract lipid R_2_ relaxation rates (Figure 3C). It was found that low microsecond motions contribute to lipid R_2_ already in the liquid phase, well above the lipid transition temperature T_m_ (the temperature at which the transition from liquid to gel is one half complete). The experiments showed that with temperature reduction, reduced lipid fluidity due to restricted rotational and segmental lipid motions is associated with an increasing amount of microsecond motions. These microsecond motions slow down further near and at its T_m_, eventually reaching the millisecond time scale below the T_m_ (Frey et al., 2018).

To investigate what impact such a behavior has on OmpX μs motions, ^15^N *R*_1_ρ experiments at different temperatures were recorded. These experiments showed that a temperature reduction from 316 to 309 K has only very little effect on OmpX's μs motions when OmpX is surrounded by DMPC14:1, since the temperature reduction is still well above the T_m_ of DMPC14:1 which is 269 K. In contrast, a strong increase of μs motions was observed for OmpX in DMPC14:0 (T_m_ = 298 K), because the temperature reduction was within the liquid-to-gel phase transition. Importantly, a temperature reduction to 293 K revealed increasing μs motions also for OmpX in DMPC14:1. Since the solvent-viscosity and all other environmental factors were similar for both samples, increased lipid microsecond motions due to decelerated lipid segmental and lipid rotational motions must be the reason for the observed differences in membrane protein microsecond motions at lower temperatures. In order to get qualitative insights into slow motions, the authors used 2D [^15^N,^1^H]-TROSY spectra as a qualitative measure for millisecond dynamics (Figure 3D). For OmpX in DMPC14:0, the authors found a strong line broadening of β-barrel resonances at lower temperature, resulting in reduced signal intensities which almost completely disappear for the 2D spectrum at 293 K. For OmpX reconstituted in DMPC14:1, the line broadening effects are temperature shifted to lower values, according to the T_m_ of DMPC14:1. Taken together, the temperature-dependent increase of μs motions and the disappearance of OmpX β-barrel resonances in DMPC14:0 above 300 K are due to increased μs–ms protein dynamics that are a result of increased μs and ms lipid motions (Frey et al., 2018).

In another study, the small multidrug transporter EmrE, an antiparallel dimer containing 4 transmembrane helices, was investigated. It is a well-studied membrane protein that is specific to a wide range of antibiotics and antiseptics (Yerushalmi et al., 1995). EmrE is an inherently dynamic transporter that converts between several conformations to transport substrates across the membrane (Yerushalmi et al., 1995). The conformational heterogeneity with an k_ex_ of ca. 500 s^−1^ at 45°C results in very poor spectra with few observable amino acid peaks (Cho et al., 2014). Upon addition of TPP^+^, a polyaromatic cation substrate, the conformational heterogeneity was slowed down and revealed two visible states to the authors, the inward- and outward-open conformation (Morrison et al., 2011). This is similar to the above mentioned PagP were the conformational exchange was slowed down by a temperature reduction. Similar to PagP, the observation of both states allows the usage of the ZZ exchange experiment. By varying the mixing time and analysis of cross and diagonal peak intensities, a global exchange rate of ca. 5 s^−1^ at 45°C was found (Miloushev et al., 2008; Morrison et al., 2011). The authors also took into account different relaxation rates of residues from a tightly packed protein environment and a loosely packed solvent-exposed environment as EmrE interconverts between the inward- and outward-open state. Interestingly, later on the Henzler-Wildman group found that the exchange rate varies with the substrate. For example, MeTPP+ has a k_ex_ of 190 s^−1^, whereas DPhTPP+ exhibits a k_ex_ of 0.4 s^−1^. The differences in rate might reflect the unique properties of multidrug recognition and transport and may occur in many other multidrug transporters (Morrison and Henzler-Wildman, 2014).

Over the years backbone relaxation experiments revealed important insights into protein dynamics. New pulse sequences even allow to quantify the populations, interconversion kinetics and structural features of sparsely populated “invisible” states and holds great promise to investigate lowly populated MP states (Vallurupalli et al., 2012, 2017). New relaxation dispersion experiments permit exchange lifetime events to be detected down to 25, 9.4 and 3.4 μs for ^15^N, ^13^C and ^1^H nuclei, respectively (Ban et al., 2012, 2013; Smith et al., 2015). This progress narrows the “hidden” supra-τ_c_ window and have the potential to provide new insights into backbone motions of membrane proteins.

## Methyl Groups as Probes for Membrane Protein Dynamics

Backbone experiments are usually limited to molecular weights below 100 kDa. Signal overlap and broad peaks due to slow molecular tumbling and chemical exchange complicates the extraction of high-quality ^15^N backbone relaxation data for larger MP-lipid assemblies and thus made their investigation considerably more difficult so far (Raschle et al., 2009). Side chain methyl groups, on the other hand, have favorable properties that permit the recording of solution NMR spectra with high sensitivity and resolution even for very large molecular assemblies with several hundreds of kDa (Sprangers and Kay, 2007b; Religa et al., 2010). Methyl groups are located at the end of their respective side chain that exhibit a higher mobility and hence a “local tumbling” that is much shorter than the overall tumbling of the protein backbone. Furthermore, magnetization for the methyl group can originate from up to three equivalent protons that rotate rapidly and are immune to solvent exchange phenomena (Tugarinov and Kay, 2005; Kay, 2011). Combined with the advent of the methyl-TROSY effect (Tugarinov et al., 2003), methyl NMR has extended the scope of NMR to biomolecules that were previously only accessible for crystallography. Another advantage is the commercial availability of precursors for specific Met, Ala, and Thr methyl labeling as well as the possibility for stereospecific labeling of either of the two methyl groups of Val, Leu, Ile. Highly selective methyl group protonation in a deuterated background reduces spin diffusion for long-distance methyl-methyl NOEs and increases the molecular weight capability of solution NMR even further. However, if the system of interest is not a homo-multimer, the assignment process of methyl groups in 100+ kDa assemblies via the backbone might not be possible and extensive mutagenesis needs to be applied. Furthermore, the costs of precursors still pose a challenge for many laboratories and commercial precursors are only available for bacterial cells, making selective methyl labeling in yeast (Dikiy et al., 2019) or insect cells (Opitz et al., 2015; Franke et al., 2018) more laborious and expensive.

The option to label the respective methyl group in bacteria as ^13^CH_3_, ^13^CH_2_D, or ^13^CHD_2_ broadens their applicability for a plethora of experiments and relaxation parameters, each with its pro's and con's (Ollerenshaw et al., 2005; Ishima and Bagby, 2018; Stetz et al., 2019). For example, fast dynamics in methyl side chains have been measured using ^2^H relaxation (Muhandiram et al., 1995). A ^3^CH_2_D group ensures a simple mono-exponential decay that is largely dominated by the ^2^H quadrupolar interaction. Five ^2^H relaxation parameters can be measured including ^2^H R_1_ and ^2^H *R*_1_ρ by using the ^3^CH_2_D group. A total of nine ^2^H relaxation rates can be measured using the ^13^CHD_2_ group (Liao et al., 2012). In a recent approach by the Palmer lab, ^2^H relaxation rate constants were measured using 400, 500, 800, and 900 MHz spectrometers and analyzed by three approaches to determine spectral density values (Hsu et al., 2018). They found that the joint-interpolated method resulted in 10–15% more precise estimates of model-free parameters from 2H spin relaxation data.

The use of ^13^CH_3_ groups significantly boosts the sensitivity for ^13^C relaxation experiments. However, due to intramethyl cross-correlated relaxation effects, these ^13^C relaxation rates are non-exponential and difficult to interpret (Igumenova et al., 2006; Stetz et al., 2019). In contrast, ^13^C relaxation rates from a ^13^CHD_2_ group are straightforward to interpret, but the absence of 2 protons and the missing methyl TROSY effect reduces sensitivity significantly (Ishima et al., 2001). Similar to the backbone order parameter for fast timescale motions, an order parameter for the methyl group was defined and denoted $S_{axis}^{2}$, describing the amplitude of motion of the bond vector about which the methyl group rapidly rotates, i.e., the bond connecting the methyl carbon and its adjacent carbon (Muhandiram et al., 1995; Kay et al., 1996).

Similar to backbone experiments, slower μs to ms side chain dynamics can be probed using CPMG experiments. Initial developments measured ^13^C-^1^H multiple quantum coherence to quantify ms motions (Korzhnev et al., 2004). This approach has the advantage that the dispersion profiles are sensitive to ^13^C and ^1^H chemical shift differences, in contrast to ^13^C single quantum (SQ) dispersion profiles which depend only on differences in carbon shifts.

Additionally, the use of ^13^C SQ CPMG experiments on ^13^CHD_2_ groups has been hampered by artifacts due to a deuterium interference effect between the one-bond ^13^C-^2^H scalar coupling and the rapid ^2^H spin-lattice relaxation. Without ^2^H decoupling, the measurement of ^13^C dispersion profiles gives erroneous high ^13^C R_2,eff_ values at low CPMG frequencies, which can be misinterpreted as exchange processes. This is a result from ^13^C-^2^H scalar coupled evolution of ^13^C magnetization in concert with ^2^H longitudinal relaxation that effectively interconverts the ^13^C multiplet components as they evolve (Rennella et al., 2016). For CPMG frequencies faster than 250 Hz, the deuterons are decoupled and the dispersion profile is flat, as expected for non-exchanging resonances. Application of a uniform 1 kHz CW ^2^H decoupling field improves the performance at low CPMG frequencies, but artifacts at higher frequencies then arise. Rennella et al. solved the problem by applying a ^2^H decoupling with variable field strength that is a function of the CPMG frequency. Thereby they could establish a robust experimental scheme for recording ^13^C dispersion profiles in ^13^CHD_2_ labeled proteins with flat dispersion profiles for non-exchanging residues over the complete range of ^13^C pulsing frequencies (Rennella et al., 2016).

**Figure 4:**
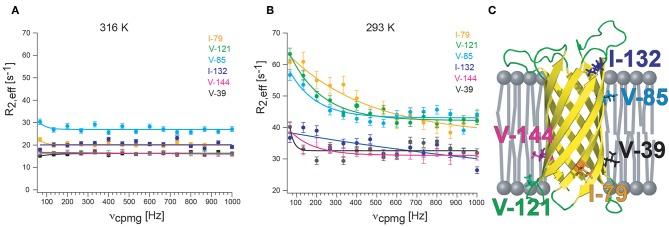
^13^CHD_2_^1^H-CPMG relaxation dispersion of OmpX in nanodiscs. **(A)** At 310 K, well above the lipid transition temperature, flat relaxation dispersion profiles are measured. **(B)** At 293 K, the transition temperature for d_54_-DMPC, relaxation dispersion curves vary strongly for different methyl groups. **(C)** Visual representation of the location of methyl groups. Methyl groups which are close to the lipid headgroup are more affected by exchange broadening than methyls outside of the lipid bilayer or located near the acyl chain ends. Since the structure of OmpX remains intact over the complete temperature range, the unequally increasing R_2,eff_ rates are likely to report on chemical exchange processes near the head group-acyl chain interface and not on conformational exchange processes. OmpX was reconstituted in MSP1D1 with deuterated d_54_-DMPC (FB reagents), containing around 80 lipids per leaflet. Only isoleucine and valines were stereospecifically labeled with ^13^CHD_2_ (NMR-Bio.com). The OmpX methyl assignment was taken from Hagn and Wagner (2015).

CPMG experiments that quantify exchange by measuring ^1^H relaxation dispersion in ^13^CH_X_ moieties can also contain significant artifacts in dispersion data sets. Artificially high ^1^H R_2,eff_ rates are due to imperfections of the refocusing pulses that lead to the interconversion of slow/fast relaxing ^1^H coherences that complicate extraction of robust exchange parameters (Korzhnev et al., 2005). The simplest way of preventing the interconversion between differentially relaxing methyl ^1^H transitions is to use a simplified ^13^CHD_2_ spin system. A sensitive pulse scheme was developed in the Kay group for recording ^1^H CPMG dispersion profiles of ^13^CHD_2_ methyl probes that could even be applied to the 360 kDa half proteasome (Baldwin et al., 2010). Recently, the Kay group could largely eliminate pulse imperfections using composite pulses phase cycled according to the XY-4 scheme (Yuwen et al., 2019). Interestingly, after applying “perfect” pulses they identified another source of artifacts in ^1^H CPMG dispersion profiles of ^13^CH_3_ methyl probes that is related to the finite length of the CPMG pulses. Because slower coherences are constantly evolved into faster relaxing elements through the action of the pulses, a different number of CPMG pulses applied for different CPMG frequencies leads to additional artifacts for large proteins were relaxation is significant (Yuwen et al., 2019). As a solution to the problem they developed a pulse sequence where the number of CPMG pulses is constant, irrespective of the CPMG frequency, eliminating the *R*_2,eff_ dependence on ν_cpmg_, that would otherwise derive from relaxation during the finite pulse widths. These efforts culminated in the first largely artifact-free methyl-TROSY based SQ ^1^H CPMG sequence for ^13^CH_3_ methyl probes (Yuwen et al., 2019). Since currently no methyl CPMG data for a membrane protein in nanodiscs or another detergent-free lipid environment are available, I provide here some preliminary and unpublished ^1^H CPMG dispersion profiles of ^13^CHD_2_-labeled methyl groups of OmpX in lipid nanodiscs. The sequence of Baldwin et al. was used (Baldwin et al., 2010) together with deuterated d_54_-DMPC (FB reagents) that is absolutely necessary to suppress the very strong natural abundance ^1^H-^13^C lipid acyl chain signals that otherwise would appear in the methyl region (Figure 4). Even with deuterated DMPC weak lipid peaks are still visible in the ^1^H,^13^C-HMQC spectrum. Well above the lipid transition temperature at 316 K, flat dispersion profiles are observed for all labeled methyl groups (Figure 4A). This is expected for OmpX that exhibits high-quality backbone and methyl NMR spectra with sharp peaks. At 293 K, the T_M_ of d_54_-DMPC (please note that deuteration lowers the transition temperature of DMPC from 298 to 293 K), dispersion profiles vary strongly for the different methyl groups (Figure 4B). V39, I132, and V144 show flat dispersion profiles. Whereas, V39 and V144 are located closer to lipid bilayer center, I132 appears to be located outside of the lipid bilayer within the solvent (Figure 4C). Much stronger R_ex_ contributions to R_2_ are found for I79, V85, and V121. These residues are located close to the lipid headgroup, near the lipid-water interface. The preliminary data are suggesting a quite complex dynamics response of the MP that depends on the position of the residue (side chain) relative to the lipid bilayer. Since the structure of OmpX remains intact over the complete temperature range, the unequally increasing R_2,eff_ rates likely report on chemical exchange processes related to the lipid-water interface rather than on conformational exchange processes.

The analysis of the different relaxation rates of double quantum (DQ) and zero quantum (ZQ) coherences (ΔR_MQ_) is an alternative route for characterizing chemical exchange processes. The group of Shimada developed a NMR method, methyl heteronuclear double resonance (methyl-HDR), to measure the ΔR_MQ,ex_ rates of methyl groups in KirBac1.1, a prokaryotic inwardly rectifying potassium channel reconstituted in DDM micelles (Toyama et al., 2016). The methyl-HDR method reduces exchange processes (ΔR_MQ,ex_) by applying spin lock fields that are stronger (i.e., faster) in field strength than the chemical exchange processes of the two scalar-coupled nuclei (^1^H and ^13^C). ΔR_MQ,ex_ rates were obtained from eight methyl groups. Methyl groups of KirBac1.1 with ΔR_MQ,ex_ rates larger than 40 s^−1^ could be found for Ile39 and Ile279, and ΔR_MQ,ex_ rates between 8.0 and 40 s^−1^ could be found for Ile138, Ile163, and Ile232 (Toyama et al., 2016). The authors conclude that these results are in line with results from crystallographic studies that showed the existence of conformational heterogeneity in the cytoplasmic region of KirBac1.1, potentially important to the allosteric regulation of the K+ permeating gate in the transmembrane region (Toyama et al., 2016).

**Figure 5:**
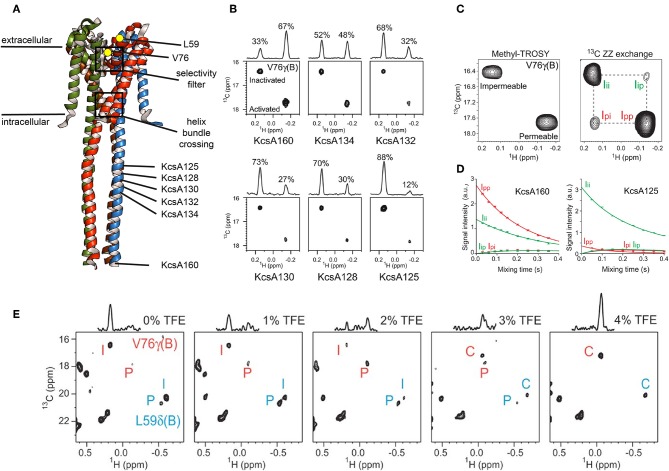
Slow conformational exchange of KcsA. **(A)** Crystal structure of KcsA in its closed conformation (pdb: 3EFF). The K^+^-ions translocate through the center of the tetramer, where the selectivity filter and the helix bundle crossing are located. Val76 and Leu59 are shown as yellow dots. Val76 is located at the selectivity filter, whereas Leu59 is located at the extracellular side of the transmembrane region. The C-terminal end of the KcsA125, KcsA128, KcsA130, KcsA132, KcsA134, and KcsA160 constructs are indicated. **(B)** Methyl-TROSY spectra of KcsA160, KcsA134, KcsA132, KcsA130, KcsA128, and KcsA125 at pH 3.2 and 40.0°C in DDM and with 50 mM K^+^. **(C)** Methyl-TROSY (left panel) and ^13^C ZZ exchange (right panel, 250 ms mixing time) spectra of KcsA160 at pH 3.2, 40.0°C, and with 50 mM K^+^. **(D)** The ZZ exchange intensities of diagonal and cross-peaks for Val76 of KcsA160 (left panel) and KcsA125 (right panel) were plotted against the mixing time at pH 3.2, 40.0°C, and with 50 mM K^+^. **(E)** Methyl-TROSY spectra of KcsA160 in nanodiscs at pH 3.2, 45.0°C, and with 200 mM K^+^ and with 0, 1, 2, 3, and 4% of TFE. P, I, and C stand for the signals from the permeable, impermeable, and closed conformations, respectively. Figure reproduced with permission from Imai et al. (2012).

Methyl ZZ exchange experiments can investigate slow conformational exchange events when methyl peaks for each conformation are visible. The group of Shimada used this type of experiment to investigate gating of the pH-dependent K^+^ channel KcsA. KcsA is a tetramer and crystal structures as well as electrophysiological studies revealed two “gates” for KcsA that are located along the K^+^ channel pathway in the center of the tetramer (Figure 5A): the selectivity filter on the extracellular side and the helix bundle crossing on the intracellular side (Imai et al., 2010). The selectivity filter is responsible for the selective permeation of K^+^ against other ions, and the helix bundle crossing interferes with K^+^ permeation. At neutral pH the helix bundle crossing is closed but opens at acidic pH, allowing K^+^ ions to flow trough. This peak K^+^ current is followed by an exponential decay to a plateau were the current is only 10–15% of the peak current. This process, called activation-coupled inactivation, cannot be explained by an opening and closing of the helix crossing bundle because it remains open at acidic pH (Imai et al., 2010, 2012). The authors demonstrated for KcsA in DDM, that the selectivity filter on the extracellular side undergoes a structural equilibrium between two distinct conformations that correspond to the permeable and impermeable states under acidic conditions (Imai et al., 2010). Both states exhibited distinct methyl peaks whose population varied with pH, temperature and available K^+^ ions (Imai et al., 2010). In a follow up study Imai et al. correlated the populations with the effect of truncation using full length KcsA (termed KcsA160) and the KcsA variants with truncated C-terminal intracellular regions (KcsA134, KcsA132, KcsA130, KcsA128, and KcsA125, Figure 5A) in DDM (Imai et al., 2012). They found that truncation changes the population of the impermeable (inactivated) and permeable (activated) state, reflected by changes of the relative intensities of the conformation specific methyl signals (Figure 5B). Subsequently, the transition rates between permeable and impermeable were measured using a ^13^C ZZ exchange experiment.

The signals of V76 for the two conformations are separated in both dimensions, making them ideal probes for a ZZ exchange experiment (Figure 5C). The dependence of the cross-peak signal intensities (I_p → *i*_ and I_i → *p*_ in Figure 5C) on the mixing time was fitted, resulting in transition rates from the permeable to the impermeable conformation of k_p → *i*_ = 0.46 ± 0.02 s^−1^ and from the impermeable to the permeable conformation of k_i → *p*_ = 0.94 ± 0.04 s^−1^ for KcsA160 (Figure 5D, left panel). For KcsA125, k_p → *i*_ and k_i → *p*_ were 7.1 ± 0.1 and 0.96 ± 0.02 s^−1^, respectively. The transition rate k_p → *i*_ is more than ten times higher for KcsA125 compared to KcsA160. The k_p → *i*_ transition rate differences as well as the population shift toward the permeable state for KcsA160 indicate that the intracellular c-terminal domain stabilizes the permeable conformation under acidic conditions when the helix bundle crossing is open. The authors also reconstituted KcsA in DMPC:DMPG nanodiscs and observed, apart from significant chemical shift changes, a striking difference in the populations. In nanodiscs, the permeable conformation of KcsA is populated around 20% (Figure 5E, left panel), whereas it is almost 100% in DDM micelles under the same acidic conditions (pH 3.2, 45°C). These results clearly show that the surrounding environment affects the conformational equilibrium of KcsA. Interestingly, the addition of TFE shifted the equilibrium first toward the permeable conformation and upon further addition of TFE toward the closed conformation, usually populated at neutral pH. It needs to be noted that a low pH can be problematic for MSP nanodiscs. The used MSP construct in the above-mentioned study, MSP1D1, has an isoelectric point (pI) of around 5.8. Titrating the solution from a pH of 7.4, were the assembly was done, down to a pH of 3.2 needs to be done with special care since MSP nanodiscs can disintegrate when going through the pI (personal experience). In another study of the Shimada group the line shapes of Methionine 82 of the β_2_-adrenergic receptor (β_2_AR) in DDM and nanodiscs were investigated and found to be different when bound to a range of agonists, indicating differences in exchange rates. By using sophisticated simulations it was found that the exchange rates between the active and inactive conformation of β_2_AR were lower in nanodiscs than in DDM micelles, and that the population of the active conformation was higher in nanodisc than in DDM micelles (Kofuku et al., 2014).

As mentioned above, the peaks for V76 and L59 of KcsA are ideal for the ZZ exchange experiment regarding their peak position and population. However, when one conformational state is so sparsely populated that there is no detectable peak, e.g., for KcsA in DDM at pH 3.2 and 45°C (Imai et al., 2010), then a methyl CEST experiment can be a good choice to reveal exchange rates and populations of the “invisible” state. An initially published ^13^C-CEST sequence used ^13^CH_3_ methyl groups and revealed an exchange between folded and unfolded conformers of a mutated SH3 domain as well as the methyl carbon chemical shifts of the invisible state (Bouvignies and Kay, 2012). Renella et al. published a ^13^C-CEST sequence using ^13^CHD_2_ methyl groups and could establish on the 360 kDa half-proteasome that this sequence is up to 5 times more sensitive than the previously published ^13^CH_3_^13^C-CEST-approach (Rennella et al., 2015). Later on the Kay lab described ^1^H CEST experiments optimized for ^13^CH_3_ and ^13^CHD_2_ methyl groups (Yuwen et al., 2017). Publications reporting on side chain dynamics of MP in a lipid environment using CEST, CPMG and ZZ exchange experiments are currently absent. However, the power of methyl groups, especially for large systems, is undisputed. Furthermore, the proximity of methyl groups to the lipid environment makes them ideal probes to measure interdependencies of MP and lipid dynamics. Environmental changes, such as temperature, lipid composition (e.g., presence/absence of cholesterol) or the measurement of a dynamic gradient of the lipid acyl chain are just a few examples were methyl probes at different depths can provide unprecedented insights into lipid-MP interactions.

## Residual Dipolar Couplings (RDC) as Probes for Membrane Protein Dynamics

The molecules of solvents used in solution state NMR generally don't adopt a preferred orientation when the sample is placed in a magnetic field. In this sense, the solvent is isotropic and proteins dissolved in isotropic solvents therefore exhibit an isotropic reorientation behavior (isotropic molecular tumbling). Interestingly, when exposed to strong magnetic fields, proteins align due to their anisotropic magnetic susceptibility. This alignment scales with the magnetic field and the proteins anisotropy of the magnetic susceptibility tensor but is usually extremely weak for small diamagnetic proteins, such as ubiquitin (Tjandra et al., 1996). However, the anisotropy of the magnetic susceptibility tensor becomes stronger for proteins containing a paramagnetic metal ion, such as cyanometmyoglobin (Tolman et al., 1995). The key feature of a residual alignment are residual dipolar couplings (RDCs). RDCs contain information on the orientation of chemically bonded nuclei relative to the magnetic field, regardless of where in the protein this bond is situated. All bond orientations are therefore restrained relative to a common frame, providing global bond-vector orientations for structure calculations that are fundamentally different from and complementary to the strictly local NOE and J coupling constraints. Unfortunately, inherent RDCs are generally so weak that they can only be measured with sufficient accuracy in favorable systems and with considerable effort (Tjandra and Bax, 1997b). It is for these reasons that substantial endeavors have been undertaken to increase the weak alignment in order to increase to the magnitude of RDCs from below 0.3 Hz (Tjandra et al., 1996) to several or tens of Hz (Tjandra and Bax, 1997a). Bicelles (assemblies composed of a mixture of short-chain and long-chain phospholipids) (Sanders and Schwonek, 1992; Sanders et al., 1994), polyacrylamide gels (Sass et al., 2000; Tycko et al., 2000; Chou et al., 2001; Meier et al., 2002; Cierpicki and Bushweller, 2004), filamentous phages (Torbet and Maret, 1979; Hansen et al., 1998; Zweckstetter and Bax, 2001), polyethylene glycol/hexanol mixtures (Ruckert and Otting, 2000), DNA nanotubes (Douglas et al., 2007; Bellot et al., 2013), macrodiscs (Park et al., 2011; Ravula and Ramamoorthy, 2019) or paramagnetic tagging (Haussinger et al., 2009; Peters et al., 2011) provide a somewhat tunable degree of alignment that promises an induction of the ideal level of alignment that is (i) large enough to easily measure RDCs, (ii) small enough to prevent spectral crowding and attenuation due to multitudes of (longrange) dipolar couplings, and (iii) small enough to preserve rapid reorientation for high-quality spectra in solution NMR. Since then RDCs have become applicable for soluble proteins (Chen and Tjandra, 2012) and intrinsically disordered proteins (Fischer et al., 2009; Mukrasch et al., 2009; Tolman, 2009; Salmon et al., 2010, 2012; Salvi et al., 2017). However, only few studies exploited RDCs for structure refinement of MP in a detergent environment (e.g., Cierpicki et al., 2006; Douglas et al., 2007; Kamen et al., 2007; Lau et al., 2008; Park et al., 2009; Berardi et al., 2011) and currently only two studies used RDCs for structure refinement of MP in a detergent free environment using lipid bilayer nanodiscs (Bibow et al., 2014; Hagn and Wagner, 2015).

**Figure 6:**
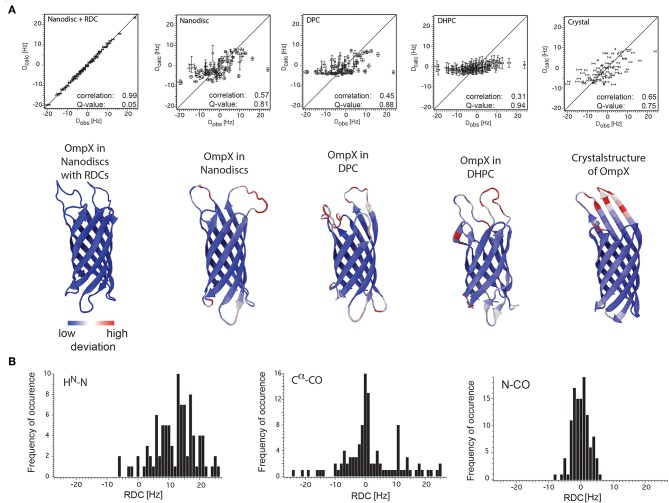
RDC values of OmpX and the nanodisc protein MSPΔH5 itself. **(A)** Correlation plots between the observed backbone amide bond RDCs and the calculated RDCs from the structures of OmpX in nanodiscs with RDCs, nanodiscs without RDCs, in DPC, in DHPC and from the crystal structure (indicated in the panels). Below each panel is the respective structure of OmpX shown with deviations from the OmpX structure calculated with RDCs (PDB: 2MNH). **(B)** RDC values of H^N^(i)-N(i), N(i)-CO(i-1), and C^α^(i)-CO(i) bonds of the ^2^H,^13^C,^15^N-labeled nanodisc protein MSPΔH5 itself measured using 10 mg/ml phages and 3D-TROSY-HNCO experiments (Yang et al., 1999; Permi et al., 2000). The use of 10 mg/ml Pf1 phages in a solution with 100 mM NaCl resulted in excellent spectra that allowed for the extraction of 97 H^N^-N, 113 N-CO and 84 C^α^-CO one-bond RDCs, ranging from −6 to +26 Hz, −8 to +6 Hz, and −24 to +25 Hz, respectively. Figure reproduced with permission from Bibow et al. (2014, 2017).

In both studies lipid-bilayer nanodiscs containing the β-barrel protein OmpX could be aligned using Pf1 phages. The resulting ^1^H-^15^N backbone RDCs were used to refine the structure of OmpX in nanodiscs and have significantly improved the accuracy of the 3D structure including, in particular, much better-defined orientations of the ^1^H-^15^N bonds (Bibow et al., 2014) (Figure 6A). Later on, it could be shown that the RDC-refined OmpX structure showed almost identical secondary structure content and orientation of β-strands when compared to the NOE-determined structure of OmpX (Hagn and Wagner, 2015). Recently, in addition to H^N^(i)-N(i) RDC, also N(i)-CO(i-1) and C^α^(i)-CO(i) RDCs could be measured of the ^2^H, ^13^C, ^15^N-labeled nanodisc protein MSPΔH5 itself. This indicates the possibility to measure these RDCs also for MP incorporated in nanodiscs (Bibow et al., 2017) (Figure 6B).

RDCs are also sensitive to motions (Blackledge, 2005; Tolman and Ruan, 2006). In an early study of cyanometmyoglobin, a paramagnetic form of myoglobin, RDCs of several Hz could be extracted. It was found that the experimentally measured values disagree with expected values calculated from available crystallographic structures. The discrepancy could be reduced significantly when slower (μs) rigid helical motions within a cone or along a one-dimensional arc were incorporated (Tolman et al., 1997). For a static protein structure without internal motions, a good agreement between the calculated (crystallographic) and experimentally (NMR) derived RDCs would be expected. However, if motions around the average position of the internuclear vector (e.g., the backbone ^1^H-^15^N bond vector) are present, they will reduce the RDC value by a factor that correlates with the amplitude of this motion. The experimentally measured RDC values were therefore dynamically averaged and can function as a probe for internal dynamics. Although the above mentioned interpretation regarding the discrepancy between measured and calculated RDCs generated considerable controversy during that time (Bax and Tjandra, 1997), it is clear now that motions between 100's of ns and 10's of μs exist and that RDCs have enormous potential to describe these motions that had until then been very difficult to access. RDCs are not widely used for membrane proteins. To the knowledge of the author no study exist which investigated MP dynamics using RDCs. Hence, I will review here studies on soluble proteins that may serve as a blueprint and provide ideas for future dynamics studies of MPs.

For soluble well-folded proteins, it is often safe to assume that the secondary structures of individual domains of the same protein are rigid whereas the relative orientation and mobility of domains can vary. RDCs are very powerful in assessing the different domain mobilities if the alignment medium has the ability to align one domain more strongly than the other. As explained by Tolman et al., this is because large scale domain motions will tend to change the overall alignment in a way that it will tend to counteract the effects of alignment (Tolman and Ruan, 2006). For example, during a structural study of the B and C domains of barley lectin, it was noted that the magnitudes of RDCs measured for each of the two domains were strikingly different (Fischer et al., 1999). This was attributed to the preferential association of the B domain with the CTAB-doped bicelles used to establish alignment. The amplitude of the dynamic sampling of domain C relative to domain B could be estimated to be on the order of 40° if a diffusion-on-the-cone model was used. In another study the domains KH3 and KH4 of the FUSE binding protein (FBP) in complex with single stranded DNA were investigated (Braddock et al., 2001). Both domains are of equal size and linked with a flexible 30-amino acid linker. However, they exhibit highly contrasting alignment characteristics when dissolved in dilute phage fd. This was ascribed to the existence of large amplitude interdomain motions and sufficient orientational independence that allows each domain to dominate their individual alignment characteristics (Braddock et al., 2001). These examples show that based on the distribution of RDCs measured for individual domains, it is possible to rapidly identify cases in which the domains are mobile relative to one another (Clore et al., 1998; Braddock et al., 2001).

The multisubunit protein β-barrel assembly machine (BAM), located at the outer membrane of Gram-negative bacteria, catalyzes the insertion and folding of the β-barrel proteins into this membrane. In *e. coli*, the BamA consists of a 16-stranded C-terminal β-barrel domain and five N-terminal periplasmic polypeptide transport-associated domains (POTRA1-5, Figure 1B). The POTRA motifs are key to BAM complex formation and interaction with the substrate β-barrel proteins. Several reports indicate flexibility between the POTRA domains resulting in compact and extended conformations (Knowles et al., 2008; Gatzeva-Topalova et al., 2010; Fleming et al., 2016). The group of Sousa used RDCs to refine the structure of POTRA4-5. Notably, a large number of the measured RDCs deviate significantly from predicted RDCs that were derived from a crystal structure. Hence, 47% of RDCs were discarded and only subset of 70 RDCs that showed good agreement (within ±5 Hz) between the experimental and predicted RDCs were used. Based on the remaining RDCs the authors concluded that the two domains behave as a single rigid species (Gatzeva-Topalova et al., 2010). It might be that the number of measured and predicted RDCs for POTRA4 and POTRA5 could increase significantly when interdomain motions or internal motions would've been allowed. The POTRA domains represent an interesting and feasible protein complex with individual domains that may be of interest to investigate how/if substrate-recognition may change interdomain flexibility. Furthermore, alignment changes of individual POTRA domains when probed with different substrate length may be useful to derive a mode of action for substrate handover (toward the BamA β-barrel) and POTRA domain reorganization. Similarly, periplasmic interdomain motions for TamA or ABC transporters may be detected and described using RDCs.

The above examples used RDCs to explain global domain motions. RDCs can also be used to probe local motions. In the Griesinger lab mathematical methods have been developed to deliver an RDC order parameter $S_{RDC}^{2}$ which describes the amplitude of motions up to milliseconds. A comparison between RDC-based order parameters $S_{RDC}^{2}$ and Lipari–Szabo order parameters $S_{LS}^{2}$ in ubiquitin reveals that $S_{RDC}^{2}$ are substantially lower than $S_{LS}^{2}$ for several residues. Since the Lipari–Szabo order parameters are only sensitive for motion faster than the molecular tumbling time τ_c_ (in the case of ubiquitin from picoseconds up to ca. 4 ns) while $S_{RDC}^{2}$ are sensitive up to milliseconds, it was speculated that additional dynamics slower than τ_c_ exist that affect $S_{RDC}^{2}$. NMR relaxation dispersion experiments detected slow timescale dynamics only for very few residues in ubiquitin. The additionally observed dynamics picked up in the $S_{RDC}^{2}$ order parameters must therefore be slower than τ_c_ but faster than 50 μs, the previously inaccessible (supra-τ_c_) time window (Lakomek et al., 2008). Subsequently, a structure ensemble was calculated that included the RDC information from 18 different alignment media. In contrast to an ensemble that was calculated using NOE data, the RDC ensemble revealed a structural heterogeneity that included all 46 ubiquitin crystal structures, which were mostly in complex with other proteins. Conformational selection, rather than induced-fit motion, thus suffices to explain the molecular recognition dynamics of ubiquitin (Lange et al., 2008).

In another study the Griesinger lab measured methyl group RDCs in 13 different alignment media in order to access and describe their ns–μs dynamics. A very wide range of motional amplitudes exist in side chains depending on solvent exposure, residue type and distance to the backbone. The authors found considerable additional dynamics slower than the correlation time τ_c_, that contributed as much mobility to the dynamics of the methyl groups as the ps-ns motion measured from relaxation data (Fares et al., 2009). The RDC-derived order parameters are dominated by rotameric interconversions and faster libration-type motions around equilibrium positions. These experiments could be of high importance for membrane proteins, especially for G-protein coupled receptors (GPCRs), where local dynamics in the 100's of ns to 10's of μs may play a role for ligand recognition and signal transduction. GPCR signal transduction is highly complex since they can signal through G-protein-dependent and G-protein-independent pathways. Biased ligands, selective for either pathway, regulate biological functions of GPCRs in a precise way (Rankovic et al., 2016). Does biased signaling for either pathway transmit trough the same (allosteric) network of residues? Local RDC dynamics using methyl groups could help to elucidate different signal transduction pathways upon ligand binding and could provide a rationale for biased agonism and future GPCR drug design.

Three main challenges can be identified that are currently limiting the use of RDCs for dynamics studies of membrane proteins. First, as pointed out by Ad Bax, dipolar couplings calculated for a distribution of vectors around an average position only start deviating significantly from the average orientation once the distribution becomes wide, that is, cone angles larger than 20° (Bax, 2003). Hence, protein backbone fluctuations of moderate amplitude are not easily detected by dipolar couplings.

**Figure 7:**
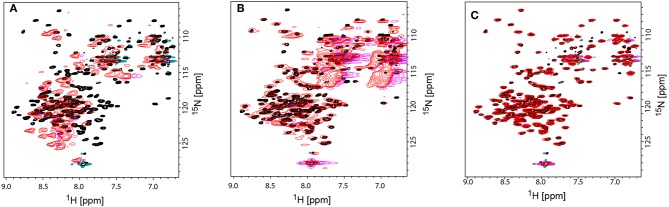
Comparison TROSY-HSQC spectra of unaligned and aligned ^15^N-labeled MSPΔH5 nanodiscs to check the structural integrity of nanodiscs. **(A)** Overlay of unaligned MSPΔH5 (black spectrum) and with C12E5/Hexanol aligned MSPΔH5 (red spectrum). Strong peak shifts in the red spectrum toward random coil values indicate the disassembly of the nanodiscs. **(B)** Overlap of peaks for the unaligned MSPΔH5 (black spectrum) and MSPΔH5 aligned in 5% neutral polyacrylamide gel indicating the applicability of polyacrylamide gels for RDC measurements. However, broad peaks most likely due to a reduced molecular tumbling, low signal-to-noise ratios and strong natural abundance ^15^NH_2_ and ^13^C = O peaks from polyacrylamide molecule disturbed peak analysis in 2D-TROSY and 3D tr-HNCO experiments, respectively. **(C)** Overlay of unaligned MSPΔH5 (black spectrum) and aligned MSPΔH5 with 10 mg/ml Pf1 phages (red spectrum) show the very good applicability of phages for RDC measurements. All samples contained 20 mM TRIS, 100 mM NaCl, pH 7.4. Negative peaks are represented in magenta and turquoise for aligned and unaligned spectra, respectively.

However, for rotameric averaging of sidechains, were the difference in orientation between rotamers is about 110°, the effect is quite large and possibly exploitable (Bax, 2003). Second, the accuracy of RDCs measured from rather large assemblies (close to or above 100 kDa) remains another major challenge. It is therefore conceivable that methyl RDCs could play a more dominant role in the future in assessing dynamics of membrane proteins in a detergent-free environment (Sprangers and Kay, 2007a; Williams et al., 2019). Their sharper signals due to their flexibility and their higher signal intensity due to 3 hydrogens per group will lead to superior RDC accuracy and quality when compared to backbone ^1^H-^15^N RDCs. The third presently limiting factor is the availability of additional alignment media suitable for RDC measurements. Currently only phages were applied successfully to align MSP-nanodiscs and their incorporated membrane protein. Studies in our lab to identify additional alignment media using empty nanodiscs failed (Figure 7). The use of ^15^N-^13^C-labeled empty nanodiscs provides a possibility to test for the structural integrity of the nanodisc itself which may be compromised upon addition of specific alignment media/compounds/proteins. DNA nanotubes and macrodics may serve as suitable alignment media for membrane proteins (Park et al., 2011; Ravula and Ramamoorthy, 2019), but have not yet been tested.

## Conclusion

Membrane proteins incorporated in lipid nanodiscs or other lipid-containing scaffolds like styrene maleic acid (Knowles et al., 2009; Dorr et al., 2014; Scheidelaar et al., 2015; Lee et al., 2016; Ravula et al., 2017b) in combination with solution NMR is very powerful in providing dynamics information with residue-resolution for the membrane protein as well as for the surrounding lipid environment (Frey et al., 2018). ZZ exchange and CPMG experiments empower the researcher with tools to investigate interconversion kinetics between and conformational plasticity of the open, closed, excited, agonist- and/or antagonist-bound state. High-power relaxation dispersion experiments permit exchange lifetime events to be detected down to 25, 9.4, and 3.4 μs for ^15^N, ^13^C and ^1^H nuclei, respectively (Ban et al., 2012, 2013; Smith et al., 2015), reducing the “hidden” supra-τ_c_ window between low μs motions and fast-timescale motions in the ns time regime. New pulse sequences, such as methyl ^13^C- or ^1^H-CEST even allow to quantify populations, interconversion kinetics and structural features of sparsely populated “invisible” protein states and holds great promise to investigate lowly populated “excited” membrane protein conformations (Vallurupalli et al., 2012, 2017). The applicability of methyl RDCs (Sprangers and Kay, 2007a; Williams et al., 2019) for the investigation of membrane protein dynamics in 100+ kDa assemblies remains to be seen. Nevertheless, all these new developments provide exciting new opportunities to investigate membrane protein dynamics in a more natural lipid environment on a wide range of timescales. The opportunity to reveal dynamic interdependencies between lipids and MP upon variation of temperature, lipids and other environmental factors is evident (Frey et al., 2017, 2018). Solution NMR and lipid nanodiscs therefore have great potential to uncover unprecedented lipid-MP dynamics relationships that allow us to explain things we have not been able to explain before.

## Author Contributions

SB wrote the manuscript and conducted the preliminary CPMG data as well as the RDC data on MSPdH5.

### Conflict of Interest

The author declares that the research was conducted in the absence of any commercial or financial relationships that could be construed as a potential conflict of interest.
